# Neural and Navigational Features Influencing the Novelty Induced Benefits on Episodic Memory

**DOI:** 10.1002/hipo.70109

**Published:** 2026-06-18

**Authors:** David A. Vogelsang, Carla Maló Calmus, Madeleine Vohs, Kreeta I. L. Kerkkänen, Svetlin Hansov, Judith Schomaker

**Affiliations:** ^1^ Faculty of Social and Behavioural Sciences, University of Amsterdam Amsterdam the Netherlands; ^2^ Programme Group Brain & Cognition, University of Amsterdam Amsterdam the Netherlands; ^3^ Medical Research Council Cognition and Brain Sciences Unit Cambridge UK; ^4^ Faculty of Social and Behavioural Sciences, Leiden University Leiden the Netherlands; ^5^ Leiden Institute for Brain & Cognition Leiden the Netherlands

**Keywords:** episodic memory, novelty, roaming entropy, spatial navigation, theta oscillations

## Abstract

Studies in animals have robustly shown that exposure to novelty can promote memory for information presented in the temporal vicinity. In humans, however, evidence for such novelty‐related memory benefits has been mixed. In this EEG study, we investigated the neurobiological mechanisms underlying effects of novelty on memory and whether individual differences in exploration patterns help explain these inconsistencies. We examined the role of theta oscillations in exploring a novel or familiar environment as well as whether spatial exploration behavior can modulate the beneficial effects of novelty on memory. Participants first explored one of two virtual environments and subsequently explored the same (familiar condition) or a new environment (novel condition). After exploring novel and familiar environments, participants performed a word learning task followed by a free recall and recognition memory test. Neurologically, exploration of the familiar rather than novel environment increased theta power, which may reflect environment‐related memory processes. However, we did not observe any differences in theta power associated with successful encoding of words after exploring a novel versus familiar environment. Behaviorally, no main effect of novelty on free recall was observed. Crucially, when accounting for variance in spatial exploration patterns, words encoded after exploring a novel environment were recalled better than words encoded after exploring a familiar environment. Furthermore, an interaction effect between the condition and exploratory behavior revealed that increased exploration benefitted free recall specifically in the familiar condition. These findings emphasize the importance of considering the way in which individuals explore a virtual environment when examining novelty effects on memory.

## 1 Introduction

Will you remember reading this article? Interestingly, findings at the cellular and molecular, but also behavioral level suggest that the fate of new memory traces is not only determined by the event itself. Neurochemical processes in the window around the event influence whether an event will be encoded into long‐term memory or not (Dunsmoor et al. 2022). Memory enhancements have been observed especially for weakly encoded events that are encountered in the temporal vicinity of a motivationally salient event. That is, events that were otherwise likely to be forgotten but that are preceded or followed by a highly salient event have a higher chance of being remembered (Duszkiewicz et al. 2019). This literature thus suggests that chances will be higher that you remember this paper if you just experienced a significant event such as witnessing a purple elephant crossing the street (a novel event) or missing your plane (an emotional event).

Although the memory facilitating effects of novelty have been well‐documented in animals, research in humans is controversial. In a first attempt to bridge the gap between the two literatures, we employed Virtual Reality (VR) to expose human subjects to spatial novelty (Schomaker et al. 2014). On a subsequent memory test, unrelated to the exploration of virtual environments, word recall was enhanced when participants explored a novel rather than a familiar environment shortly before memory encoding. In follow‐up studies, we identified several limitations to these effects. For example, novelty‐induced memory enhancements were only found when individuals actively explored a novel environment, and not when they were passively exposed to the exploration behavior of someone else (Schomaker and Wittmann 2021).

Beneficial effects of novelty on memory have been observed in a scholastic environment (Ballarini et al. 2013; Ramirez Butavand et al. 2020) and for different types of learning material, including verbal information (Fenker et al. 2008; Schomaker et al. 2014; Schomaker and Wittmann 2021; Baumann et al. 2020), visual information (Abrahan et al. 2020; Ballarini et al. 2013) and motor learning (Ruitenberg et al. 2022). However, some recent studies failed to find novelty‐related memory benefits (Quent and Henson 2022; Biel et al. 2020; Biel and Bunzeck 2019; Servais et al. 2026; Raza et al. 2023). In a recent review we introduced a theoretical framework, suggesting that novelty‐induced memory enhancements depend on several crucial factors, including the stimulus type, timing and involvement with the novel material (Lorents et al. 2023). Although our framework can explain some of the discrepant findings, other contradicting results could not be accounted for, and it thus remains unclear what the optimal conditions for novelty to promote memory are. The scientific challenge of identifying these conditions may be related to a gap in the literature linking behavior and underlying neural mechanisms into one unified framework. It remains unclear how exploratory behavior in novel environments interacts with the neural oscillatory processes that are hypothesized to modulate novelty‐related memory benefits.

A potential explanation for these discrepant findings lies in the fact that previous studies inducing novelty through (virtual) environments have not accounted for individual differences in exploration behavior, an aspect that may critically shape both the behavioral benefits of novelty on episodic memory and the neural mechanisms underlying novelty processing. Research on hippocampus‐dependent navigation highlights that actively exploring novel environments is crucial for building cognitive maps, which support efficient navigation in both non‐human animals and humans (Epstein et al. 2017; Bellmund et al. 2018). This active exploration allows for the integration of multisensory information necessary for forming a comprehensive and flexible representation of the environment within the hippocampus (O'Keefe and Nadel 1978). Cellular hippocampal mechanisms related to the exploration of a novel environment have in turn been shown to be crucial for the beneficial effects of novelty on memory in animal studies (see, e.g., Moncada and Viola 2007). However, prior research on novelty effects has yet to link spatial behavior to memory benefits during episodic encoding. From a theoretical perspective, these patterns are critical to consider, as exploration metrics, such as total path length and time spent exploring landmarks, may influence the strength of novelty‐induced memory effects by differentially engaging hippocampal processes. While Schomaker et al. (2022) did compare navigation behavior in novel and familiar VR environments and found higher roaming entropy, quantifying exploratory behavior, in familiar environments compared to novel ones, it was not investigated whether variance in roaming entropy could explain novelty‐related memory benefits. As previous studies failed to take individuals' exploration behavior into account when investigating the effects of novelty on memory, it is possible that such differences contributed to the equivocal results in the literature. It thus remains unclear how spatial exploration patterns interact with novelty to influence subsequent free recall, underscoring the need to bridge this gap in the current literature.

Another factor that has received limited attention in research on novelty‐induced memory benefits in humans, is the role of neuronal oscillatory mechanisms underlying these effects. Examining these mechanisms in tandem with behavioral measures of exploration may provide a more complete account of how novelty enhances (episodic) memory. Studies in rodents have suggested that two different systems are driving the effects of novelty on memory, through their effects on hippocampal dopamine (Duszkiewicz et al. 2019; Takeuchi et al. 2016; Kempadoo et al. 2016; McNamara and Dupret 2017): (1) Novelty‐related activation of the locus coeruleus (LC) has been observed after the learning event, while (2) ventral tegmental area (VTA) activation has beneficial effects on hippocampal memory during learning. Furthermore, in a seminal paper, Lisman and Otmakhova (2001) suggested with their SOCRATIC model that novelty brings the hippocampus in a learning, rather than a recall mode. This learning mode has theoretically been linked to theta oscillations in the brain, but there currently are no experimental EEG studies that have identified such a *novelty state* during exploration. The animal literature thus has provided a scope by which the effects of novelty on memory can play out, but the neural mechanisms of exploring a novel environment and their subsequent effect on memory have not yet been addressed in humans.

Theta oscillations have been extensively linked to navigation and memory processes, with prior work emphasizing their pivotal role in spatial navigation (Kahana et al. 1999; Kaplan et al. 2012; Buzsáki and Moser 2013; Bush et al. 2017; Du et al. 2023) and successful encoding and retrieval of information (Herweg et al. 2020; Rudoler et al. 2022). Classic studies in rodents demonstrated the importance of theta oscillations in navigation, particularly in the firing patterns of hippocampal place cells (O'Keefe and Lynn 1976; O'Keefe and Burgess 2005). Moreover, previous research has highlighted the importance of hippocampal‐prefrontal interactions during navigation (García et al. 2025). Lesions in the medial prefrontal cortex (mPFC) of rodents disrupt spatial learning, while neural firing patterns in the mPFC encode cognitive features critical for the implementation of efficient navigation strategies (Patai and Spiers 2021). In line with this, Chrastil et al. (2022) found that active as opposed to passive exploration of a spatial environment was associated with increased theta power over frontal electrode sites. Interestingly, recent human research has suggested that memory (i.e., simulating a previously navigated route) may be a more important source of hippocampal theta oscillations than navigation itself (Seger et al. 2023). When it comes to theta and novelty effects, Lee and colleagues (2014) found that novelty as induced in an oddball task is associated with increased theta power, and Demiralp and colleagues (2001) reported that the P3a component, elicited by novel stimuli, generates substantial theta activity across distant brain regions. Other prior research has established a relationship between theta oscillations and successful encoding of episodic information (Osipova et al. 2006; Khader et al. 2010; Hanslmayr et al. 2011; Vogelsang et al. 2018). For example, research by Hanslmayr et al. (2011) showed that theta power increases during successful encoding of words. Taken together, these findings suggest a role for hippocampal and frontal theta oscillations in spatial behavior and episodic memory. However, the role of theta oscillations during successful memory formation after exploring a novel environment remains to be investigated, and doing so requires an integrated approach that considers both neural oscillatory activity and individual differences in exploration behavior.

In the current study, we aimed to examine whether behavior during free exploration, that is, the task typically used in the novelty‐related memory paradigms, of novel versus familiar environments can help explain the novelty‐induced effect on episodic memory, as well as the role of theta oscillations in supporting this effect. The first aim of this study was to examine the role of theta power during exploration of a novel versus familiar environment, with respect to which we predicted that exploration of a familiar environment would elicit increased theta power compared to exploration of a novel environment (Kaplan et al. 2012). Secondly, we wanted to replicate the behavioral prediction of prior work highlighting that recall of a list of words after exploration of a novel environment is significantly enhanced compared to recall of a list of words after exploration of a familiar environment (e.g., Schomaker and Wittmann 2021). We focused on free recall because this task strongly depends on hippocampal processes, which are known to be engaged by novelty manipulations (Eichenbaum et al. 2007; Schomaker et al. 2014). In contrast, recognition memory can often be supported by extrahippocampal regions such as the perirhinal cortex, and therefore novelty effects are not necessarily expected in recognition performance (Eichenbaum et al. 2007). Crucially, we aimed to account for individual differences in spatial exploration behavior—as measured with roaming entropy—to determine whether differences in this behavior influence the novelty effect. Lastly, we sought to examine whether improved recall of words following novel versus familiar exploration is associated with increased theta power during the successful encoding of those words. By jointly examining navigation behavior and oscillatory dynamics, this study sought to bridge previously separate lines of research to clarify how exploration and neural activity jointly contribute to novelty‐induced memory effects.

To address these questions, we conducted a three‐day EEG study combining virtual navigation and episodic memory tasks. On Day 1, participants first explored one of two virtual environments (the familiarization phase), followed by an exploration of either the same environment (familiar condition) or a new environment (novel condition). After the second exploration, participants then completed a word learning task, followed by immediate free recall and old/new recognition memory tests. On Day 2, they returned for a delayed memory test (free recall and recognition) of the Day 1 word list, then explored either the same environment that was explored during familiarization (familiar condition) or a new one (novel condition), and completed a new word learning task with its corresponding immediate free recall and recognition memory tests. Finally, on Day 3, participants performed the delayed recognition memory test for the Day 2 word list. EEG data was collected on Day 1 and Day 2 during the exploration of the virtual environemtns as well as the memory task. This design allowed us to assess both immediate (within‐session) and delayed (24 h later) memory performance, providing a means to examine the persistence of novelty‐induced memory effects and the role of theta oscillations in the novelty effect.

## 2 Methods

### 2.1 Participants

A total of fifty‐eight healthy adults participated in this experiment. Two participants were excluded from analyses due to missing EEG data, resulting in a total of 56 participants being included in the memory analyses (40 females and 16 males; mean age = 20.8 years, SD = 3.9 years, range 18–31 years; 40 right‐handed; 15 left‐handed [1 missing value]). For the exploration EEG analysis, 5 participants were excluded either due to technical errors during data collection or excessive motion artifacts, leaving a sample of 53 participants (mean age = 20.7 years; range 18–31 years). For the episodic memory encoding task EEG analysis, 14 participants were excluded because of technical errors during data collection or excessive motion artifacts, leaving a sample of 44 participants (mean age = 20.4 years, range 18–31 years). All participants had normal or corrected to normal vision and were recruited through the Leiden University participant pool. Written consent was obtained prior to the start of the experiment and participants received either €24 or course credits for their participation. Inclusion criteria for the study comprised an age range between 18 and 45 years, no usage of any drug or psychoactive medication, and no history of neurological or psychiatric disorders. The study was approved by the Psychology Research Ethics Committee (CEP; reference number: 2021‐11‐12‐J.Schomaker‐V2‐3522) of the Faculty of Social and Behavioral Sciences, Leiden University.

### 2.2 Stimuli & Materials

**FIGURE 1:**
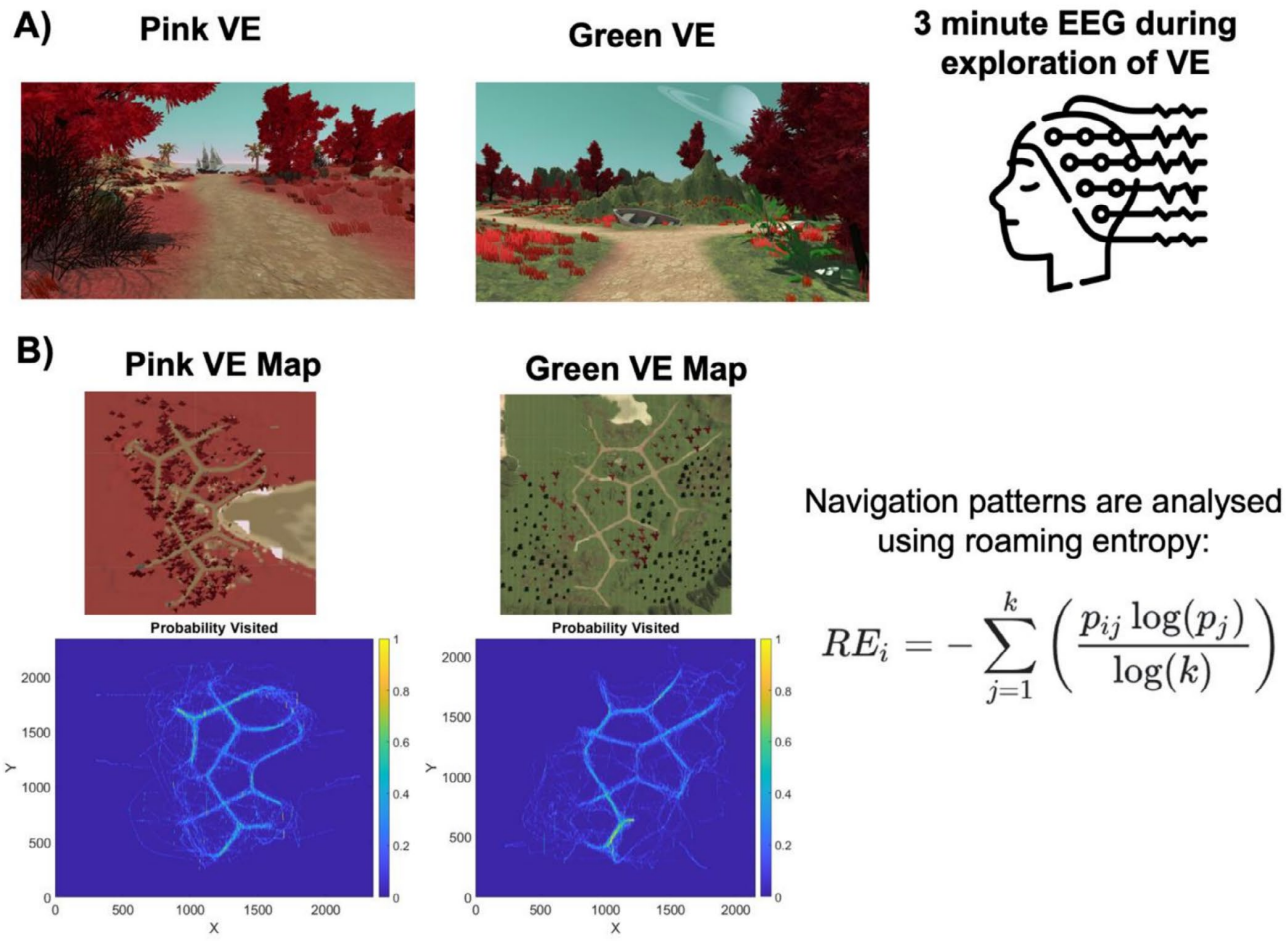
(A) Top‐view of the pink (left) and green (right) virtual environment (VE). Participants freely explored the virtual environment whilst we collected EEG data. (B) Maps for the pink and green VE depict the probability that each location was visited across participants. These maps were used in the roaming entropy calculations. Note, the maps are scaled for graphical purposes.

The Virtual Environments (VEs) used for the exploration task (see below) were created with Unity Version 2017.2.21f1 (Unity Technologies, 2017). The VEs were matched in terms of size, path length, and number of intersections. Both VEs featured imaginative islands with unique landmarks (such as an airplane) positioned at intersections or road endpoints. These islands encompassed both land and a body of water (see Figure 1 for a top‐view of the two VEs). Participants could move forward by pressing the W key on the keyboard and determine their heading direction using the mouse. Throughout the exploration, the *X*, *Y*, and *Z* coordinates of the moving agent were recorded, with a sampling rate of approximately 15 Hz.

The episodic memory encoding and retrieval tasks were created using E‐Prime 3.0 software (Psychology Software Tools, Pittsburgh, PA). For the episodic memory task, 240 words were selected from the MRC Linguistic Database and were split into two lists of 120 words, with one list being presented on day 1 and the second on day 2. Testing on the various days occurred at the same time of the day to control for variations in cognitive performance across the day. On day 1, participants additionally filled in a demographics questionnaire, the novelty seeking subscale of the Tridimensional Personality Questionnaire (Cloninger et al. 1991; Cloninger et al. 1993), some questions with regard to gaming and the BIS/BAS scales. After each exploration round (day 1–2) participants filled out the Igroup Presence Questionnaire (IPQ; Schubert et al. 1999). Participants also performed a landmark memory test to assess item memory for the landmarks encountered during the exploration task, however, this task is not of interest in this paper and will not be further analyzed and discussed here.

### 2.3 Exploration Task

Both on day 1 and day 2, participants started with a 3‐min eyes‐closed resting state EEG (rsEEG; not reported here). After the rsEEG, participants performed an exploration task, in which they were instructed to freely move around in the VE, using the W‐key on the keyboard to move forward and the mouse to determine heading. The exploration task lasted 3 min whilst continuous EEG data was collected. On day 1, participants performed the exploration task twice. The first exploration constituted the “familiarization” round, followed by participants either exploring the same environment (referred to as the “familiar” condition) or a new environment (referred to as the “novel” condition). On day 2, participants only performed one exploration task (which was either familiar or novel to them). The order of “familiar” versus “novel” on day 1 versus 2 was counterbalanced between participants. Exploration behavior was quantified with roaming entropy (RE). RE was calculated per participant (RE) for each exploration round (novel; familiar) by summating over the product of the individual's trajectory (pij) and the log probability that each location was visited (pj) divided by the log of the total number of possible locations (*k*; see Figure 1B). Higher RE reflects more exploratory behavior, indicating that an individual went more off the mostly visited paths, while lower RE reflects less exploratory behavior (e.g., when an individual stayed on the paths). RE in the novel and familiar conditions was compared with a paired *t*‐test.

### 2.4 Episodic Memory Task

After the novel and familiar exploration task, participants performed an episodic memory task consisting of a study (i.e., encoding), distractor (simple numerical calculation task), and test (i.e., retrieval) phase. During the memory encoding phase, participants were presented with 60 words (i.e., nouns) and judged whether each word described something living or non‐living. Participants had 2 s to respond with two button options (letters “X” and “N” on the keyboard) with a 1 s intertrial interval. Following the encoding phase, participants engaged in a distractor task consisting of a series of nine straightforward math problems. These problems involved basic numerical operations such as subtraction (e.g., 4–3) or addition (e.g., 7 + 1), with the solutions falling within the range of 1 to 9. After the distractor task, participants performed a free recall test in which they were instructed to recall and write down as many words as they could remember from the encoding phase. After the free recall test, participants performed an old/new recognition memory test with 60 old and 30 new words, and participants were instructed to judge whether the word on the screen was either old (pressing key “X”) or new (pressing key “N”). Words were presented for 2000 ms on the screen with a 1000 ms fixation cross in between trials.

In total, the study consisted of three sessions conducted on three consecutive days. On Day 1, participants completed the exploration task followed by the episodic memory task (encoding, distractor, and immediate free recall and old/new recognition memory test). On Day 2, participants began with a delayed free recall and recognition test of the word list studied on Day 1. They then completed a second exploration task, studied a new word list, and following the distractor task, performed the immediate free recall and recognition memory tests for this Day 2 list. On Day 3, participants returned for a delayed free recall and recognition memory test of the Day 2 word list. This design allowed us to assess both immediate memory performance (within the same session) and delayed memory performance (across a 24‐h interval).

### 2.5 Word Memory Analyses

To investigate the effect of the time of measurement and the novelty of the explored environment on word recall and recognition (as measured by corrected hit rate [CHR], that is, the difference between the hit rate and the false alarm rate) for the different order conditions, we performed two mixed ANOVAs with time (immediate; delayed) and novelty (novel; familiar) as within‐subjects factors and order of the environments (Green‐Pink‐Green; Green‐Pink‐Pink; Pink‐Green‐Pink; Pink‐Pink‐Green) as between‐subjects factor. We included order to accommodate for an unbalanced design. Effects including the factor order, were further investigated with follow‐up ANOVAs per order (see S2).

We did not observe the expected effect of novelty on memory for all orders. To further investigate this null effect we performed Bayesian statistics, comparing a model including novelty with a null model only including a time factor (see S3).

### 2.6 Moderation Analysis

To examine whether the relationship between the type of condition (novel, familiar) and free recall is moderated by spatial exploration behavior in the virtual environment, we ran linear mixed effects models with “condition” and “roaming entropy” as predictors and with “free recall” (both immediate and delayed) and “recognition memory/CHR” (both immediate and delayed) as outcome variables. Thus, we tested four models: for (1) immediate recall, (2) delayed recall, (3) immediate recognition memory/CHR, and (4) delayed recognition memory/CHR. We centered the predictors as they were on different scales, and applied a false discovery rate (FDR) correction to correct for multiple comparisons across the models. Importantly, these were within‐subject linear mixed‐effects models, as each participant completed both the novel and familiar conditions, with subject included as a random intercept, accounting for the non‐independence of the measurements across the conditions. The strength of significant effects after the multiple comparisons correction was further examined with effect sizes, specifically partial eta squared. Due to technical errors during data collection, we only collected roaming entropy data for 37 of the participants, hence this moderation analysis was only conducted on this sample of participants.

### 2.7 EEG Data Acquisition

EEG data was collected using a 32‐electrode channel cap according to the 10–20 system using a Biosemi ActiveTwo system. Additional electro‐oculography (EOG) electrodes were used to detect eye‐movement related activity that could later be used in the ICA to detect eye‐blink artifacts. Horizontal eye movements were recorded at both external canthi and vertical eye movements were monitored with one electrode below and one above the left eye. Furthermore, two mastoid electrodes were placed that were used for referencing (see EEG Analysis section). Prior to the start of the experiment, participants were instructed to minimize eye movements, blinking, and muscle movement.

### 2.8 EEG Preprocessing

All EEG analyses were conducted in MNE‐Python (Gramfort et al. 2014), ran on Python version 3.10.11. Raw EEG data was first downsampled to 250 Hz and bandpass filtered with a 0.5 Hz high and 48 Hz low pass filter. EEG data was referenced to the two mastoid electrodes and epoched. For the exploration EEG data, we epoched the continuous 3‐min EEG data with epoch length of −500 to 2000 ms. For the memory encoding task, epochs were of length −1000 to 2000 ms. ICA (picard) was used to detect EEG artifacts, and IC components that identified excessive artifacts were removed. Furthermore, any ICA components that significantly correlated with EOG activity were automatically identified and rejected by an algorithm implemented in the MNE‐Python software. Finally, all epoched EEG data were visually inspected and trials that contained excessive noise were removed, resulting in an average of less than 5% of trials removed across participants with a mean trial removal of 1.6 trials (range 0–5 trials).

### 2.9 EEG Exploration Data Analysis

For the EEG data obtained during exploration, power spectra were calculated for all channels, using Welch's method (2‐s windows, 50% overlap), and the power spectrum was computed across a frequency range of 2 to 30 Hz. Because spectral power can be influenced by broadband (1/f) activity, we decomposed each power spectrum into periodic and aperiodic components using the specparam (formerly known as FOOOF) toolbox (Donoghue et al. 2020). This step ensured that any observed differences in oscillatory power reflected true rhythmic activity rather than shifts in the aperiodic background. We used the specparam tool to parameterize neural power spectra, fitting a model to the power spectrum that captures both periodic oscillatory components (such as alpha or beta rhythms) and the non‐oscillatory aperiodic background. It provides parameters such as center frequency, amplitude, and bandwidth for each identified peak, as well as values for the exponent and offset of the aperiodic component (to remove 1/f background “noise” from the EEG data). We extracted the exponent and offset parameters provided by specparam for each exploration condition (familiarization, novel, and familiar) per participant to examine differences between conditions as a control analysis. To compare relative power differences in the theta band between the three exploration conditions, we removed the aperiodic signal from the periodic signal, resulting in a flattened power spectrum. From this flattened power spectrum, we extracted relative theta power from 5‐7 Hz in the three electrode sites of interest: frontal (Fz), central (Cz), and posterior (Pz) for each condition separately. Based on prior literature, we were mainly interested in frontal midline theta (e.g., Chrastil et al. 2022), and we therefore contrasted this frontal theta power directly with central and posterior electrodes. For the theta power, we then ran a 3 by 3 repeated measures ANOVA with the factors condition (familiarization, novel and familiar) and electrode (Fz, Cz, and Pz). Given that EEG variables often violate the assumptions of normality, we decided to determine statistical significance via a non‐parametric permutation testing approach (see for further details below).

### 2.10 EEG Memory Task Data Analysis

We were interested in the role of theta power during successful encoding of words that were subsequently recalled (i.e., free recall task) or recognized (i.e., old/new recognition memory task) after exploration of a novel or familiar environment. To compare successful encoding of novel versus familiar trials, we epoched the trials of the encoding task according to whether a word was subsequently remembered or not in the free recall and the old/new recognition memory test. Similar to the exploration data analysis, EEG analysis of the memory task data focused on central, cortical electrodes Fz, Cz, and Pz. Time‐frequency analysis was conducted using a Morlet wavelet using the tfr_morlet function as implemented in MNE‐Python with 5 cycles in a frequency range of 2–40 Hz with steps of 1 Hz between each wavelet frequency. Baseline correction was applied using the [−700, −200] ms time window prior to stimulus onset. During free recall, we observed low trial numbers for several participants (i.e., below 10 trials) and for this reason a within‐subject analysis was not feasible. Therefore, we conducted a between‐subject analysis on the free recall EEG task data, whereby participants were compared as groups (e.g., novel vs. familiar condition) rather than regressing small subsamples against behavioral outcomes. Those participants with trial numbers lower than 10 were excluded. Mean trial numbers for the encoding task for words that were successfully subsequently recalled were the following: Novel condition recalled mean trial number = 14, range 10–27; Familiar condition recalled mean trial number = 14, range 10–25. Because this was the only between‐subject analysis in our study, it is important to note the sample size per group: 25 participants had at least 10 trials in the familiar condition and 27 in the novel condition. In contrast, all other behavioral and EEG analyses reported in this manuscript were conducted within subjects. Mean trial numbers for the encoding task for words that were successfully subsequently recognized were the following: Novel condition successfully recognized mean trial number = 51, range 26–60; Familiar condition mean trial number recognized = 52, range 35–59. For statistical testing, we extracted theta power in the 5–7 Hz range and the 200–1000 ms time window and ran a 2 by 3 repeated measures ANOVA with factors condition (novel, familiar) and electrode (Fz, Cz and Pz) for both the successful subsequent recall as well as the successful subsequent recognition analysis. Given that the EEG data violated the assumptions of normality, statistical significance was determined via a non‐parametric permutation testing approach (see below).

### 2.11 Permutation Testing

Prior to the statistical analysis of the EEG exploration and memory encoding task data, we noticed that the EEG variables were not normally distributed. We therefore decided to opt for a non‐parametric approach by creating a set of critical F‐values through permutation testing (see Vogelsang et al. 2018 for similar procedure). For each contrast of interest, we first ran a repeated measures ANOVA to obtain the *F*‐value for each specific contrast. Second, we shuffled the conditions randomly for half of the participants such that pseudoconditions were created in which systematic differences between the conditions were eliminated. For these pseudoconditions, we obtained the *F*‐value for each contrast and this procedure was repeated 1000 times with every iteration a new set of participants whose data was shuffled. This provided a distribution of *F*‐values of which we took, using an alpha level of 0.05, the 950th value as our critical cut‐off F‐value. This non‐parametric approach allowed us to address the non‐normality of the data by creating our own F‐value distribution from shuffled data.

## 3 Results

### 3.1 Memory Results: Word Recall and Recognition

To examine the effect of novelty on free recall, we ran a mixed ANOVA with time (immediate; delayed) and novelty (novel; familiar) as within‐subjects factors and order of environment (Green‐Pink‐Green; Green‐Pink‐Pink; Pink‐Green‐Pink; Pink‐Pink‐Green) as between‐subjects factor. Memory performance was higher for immediate than for delayed recall, F(1, 47) = 36.95, *p* < 0.001, ŋ2 = 0.44. No main effect of novelty was observed, F(1, 47) = 0.80, *p* = 0.38, ŋ2 = 0.017, and novelty and time did not interact, F(1, 47) = 1.92, *p* = 0.17, ŋ2 = 0.039. However, a two‐way interaction between novelty and order was found, F(3,47) = 3.03, *p* = 0.039, ŋ2 = 0.162. Although no main effect of order was found, F(3,47) = 1.15, *p* = 0.34, ŋ2 = 0.07, time, novelty and order interacted, F(3,47) = 7.58, *p* < 0.001, ŋ2 = 0.33. The role of order was further investigated with separate ANOVAs (see S2). To further examine the null effect of novelty, we conducted follow‐up Bayesian statistics to find supporting evidence for the null hypothesis. These additional analyses indeed confirmed that there was no supporting evidence for a novelty effect (See Supporting Information).

We also performed a mixed ANOVA for word recognition, as measured by the corrected hit rate (CHR). Word recognition was higher for immediate than for delayed recall, F(1,52) = 8.37, *p* = 0.016, ŋ2 = 0.14. No main effect of novelty was observed, F(1,52) = 0.14, *p* = 0.66, ŋ2 = 0.004, nor of order, F(3,52) = 1.37, *p* = 0.26, ŋ2 = 0.073. Novelty and time did not interact, F(1,52) = 0.17, *p* = 0.68, ŋ2 = 0.003, nor was a three‐way interaction between novelty, time and order observed, F(3,52) = 0.06, *p* = 0.98, ŋ2 = 0.004.

**FIGURE 2:**
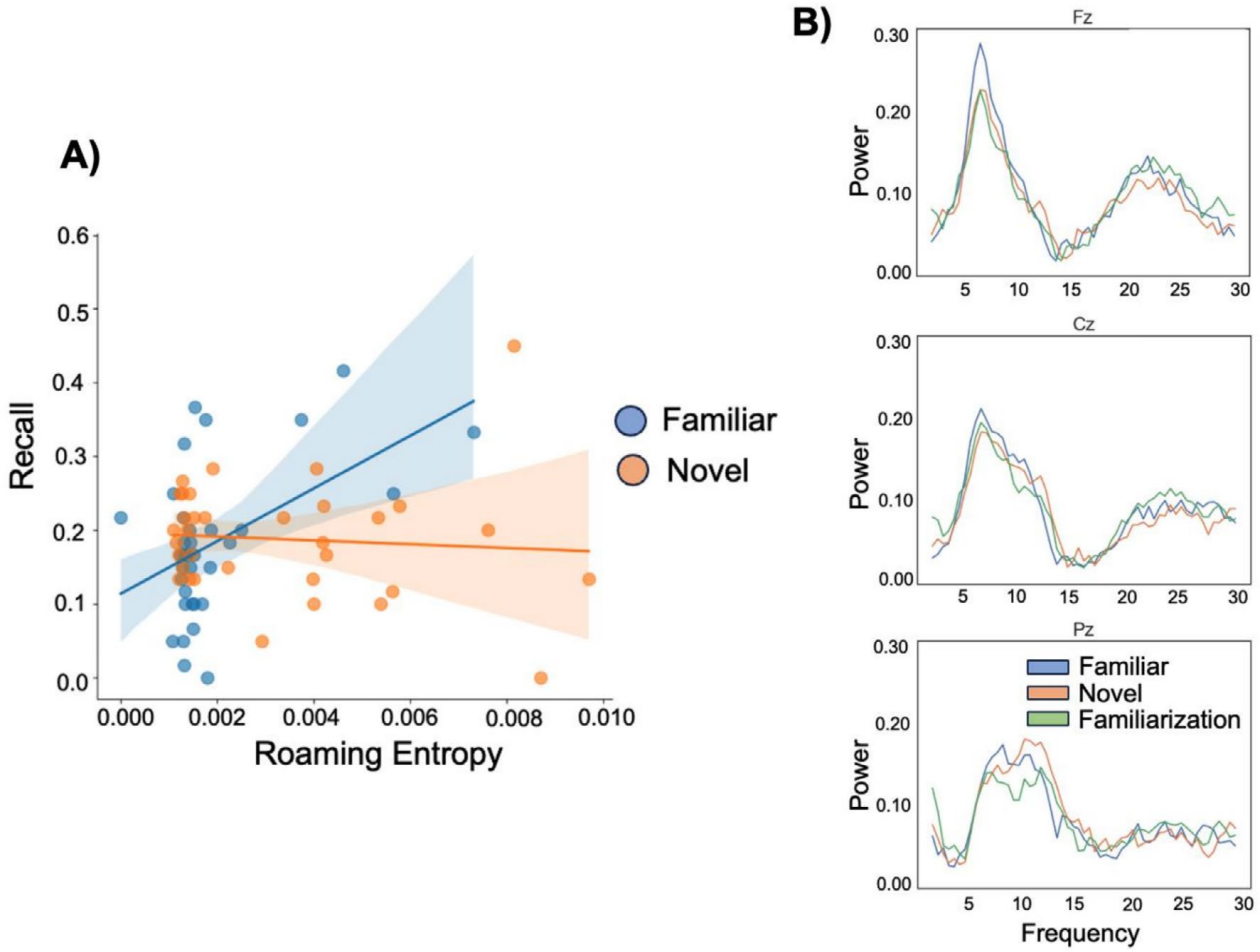
(A) Within‐subject linear mixed effects model examining the relationship between roaming entropy and free recall (i.e., immediate recall). Overall, accounting for roaming entropy, a novelty effect was observed as well as an interaction between roaming entropy and condition (familiar/novelty). For visualization purposes, we plot the results with the three outliers for roaming entropy in the novelty condition excluded. (B) EEG results of the exploration data for the familiar, novel and familiarization conditions.

We then examined whether individual differences in exploration behavior (as quantified by roaming entropy) influenced the effect of novelty on free recall. To examine this, we ran a within‐subject linear model that included “condition” (novel, familiar) and “roaming entropy” as fixed effects and a random intercept “subject” to account for individual differences in baseline free recall. We ran this model for both the immediate and delayed free recall as well as CHR data. For immediate recall, there was a significant main effect of novelty on recall performance (*β* = −0.385, SE = 0.13, *t* = −3.08, *p* = 0.03; FDR‐corrected), which was a large effect considering the corresponding partial eta squared of 0.15 (95% CI = [0.03, 1.00]). No significant novelty effect was observed for neither the delayed free recall (*β* = −0.287, SE = 0.14, *t* = −2.1, *p* = 0.10) nor CHR (immediate: β = 0.835, SE = 0.36, *t* = 2.33, *p* = 0.08; delayed: *β* = −0.23, SE = 0.35, *t* = −0.65, *p* = 0.66). Furthermore, for immediate free recall we also observed a significant novelty by roaming entropy interaction (*β* = −1.84, SE = 0.58, *t* = −3.19, *p* = 0.03), which again was an effect with a large effect size (partial eta squared = 0.15, 95% CI = [0.04, 1.00]), suggesting that the relationship between roaming entropy and free recall differed per condition. Illustrated in Figure 2A, in the novelty condition there was no clear relationship between roaming entropy and free recall, whereas in the familiar condition there was a positive relationship between roaming entropy and free recall. Making sense of this pattern, we hypothesized that in the familiar condition, greater exploration during the second visit may reflects the discovery of “novel” areas that were not explored during the initial familiarization. Consequently, increased exploration in the familiar environment may mimic a novelty effect, leading to a positive relationship between exploration and free recall. To examine this tentative hypothesis that exploring previously unexplored areas within familiar environments influenced memory, we conducted an exploratory analysis calculating the overlap between the path taken during the initial visit (familiarization) and the path during the second exploration of the same environment. When overlap was accounted for, both the interaction of novelty and RE (*β* = −27.380, SE = 14.757, *t* = −1.855, *p* = 0.247; FDR‐corrected) as well as the main effect of novelty (*β* = 0.057, SE = 0.028, *t* = 2.017, *p* = 0.211) were no longer significant. This result suggests that overlap in navigation patterns might be a key factor driving the interaction between condition (novelty, familiar) and roaming entropy on free recall. We discuss the implications of these findings in the discussion.

As an additional test of this interpretation, we examined whether the change in roaming entropy between the familiarization and familiar exploration phases predicted free recall performance. Specifically, we computed a difference score reflecting the change in exploration behavior (RE_change = RE_familiar—RE_familiarization) and tested its association with free recall. This analysis revealed a non‐significant relationship (estimate = 0.032, *t* = 0.079, *p* = 0.938).

### 3.2 EEG Exploration Results

**FIGURE 3:**
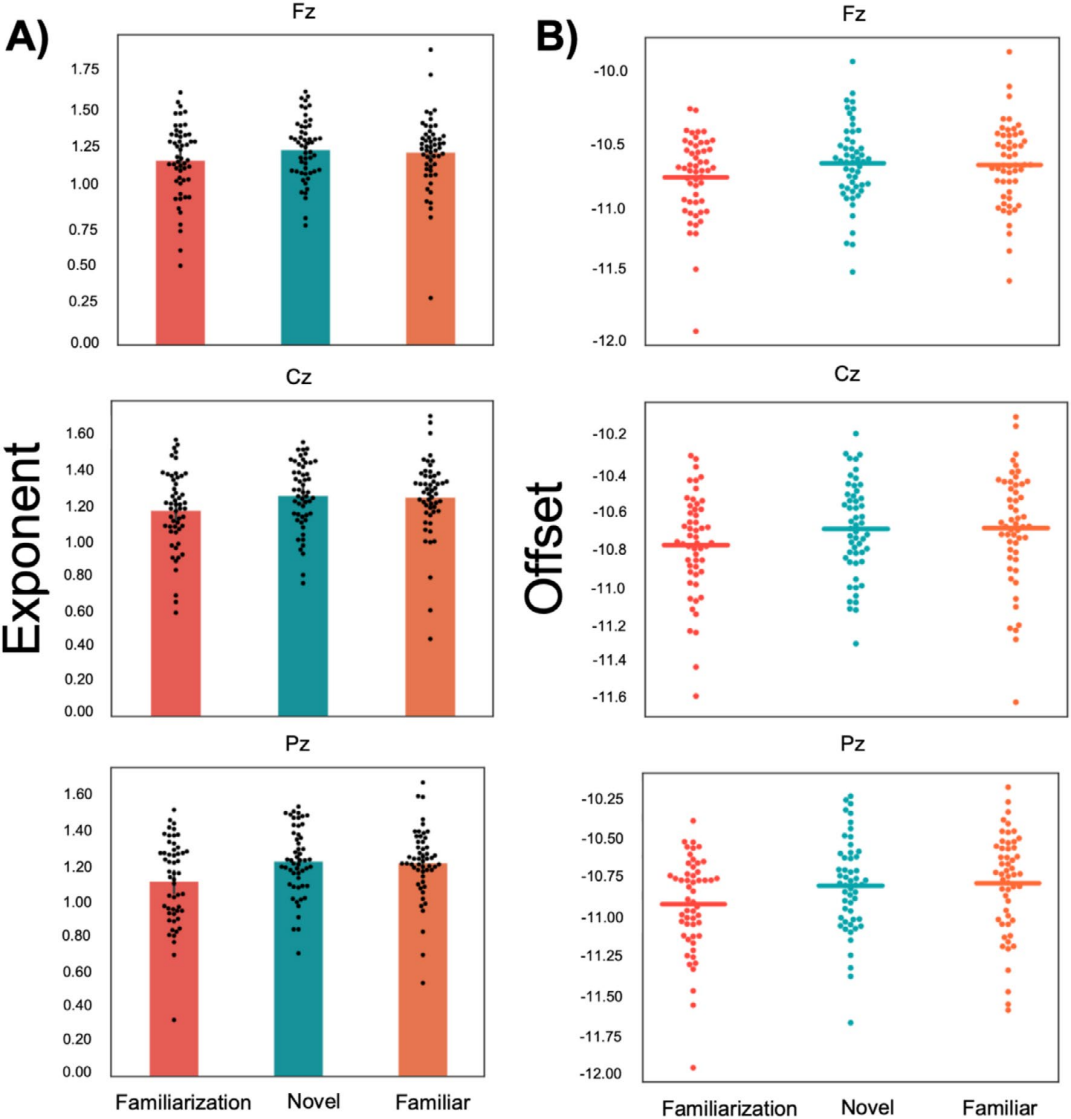
The aperiodic results during exploration of the virtual environments. (A) For the exponent, a repeated measures ANOVA with factors electrode (Fz, Cz, and Pz) and condition (familiarization, novel and familiar) revealed a main effect of condition (the familiarization condition had a lower exponent across the three electrode sites compared to the novel and familiar conditions). (B) No reliable differences were observed for the offset value between conditions.

We first examined differences in aperiodic activity between the three exploration conditions (familiarization/novel/familiar; see Figure 3). Differences in aperiodic activity, reflected in 1/f or scale‐free dynamics, may confound analyses when comparing theta power across experimental conditions (Donoghue et al. 2020). For example, differences in theta power between conditions could be due to a difference in the slope (i.e., exponent) or the offset of neuronal firing rather than true theta power differences. Therefore, isolating the periodic signal from aperiodic components can provide a more accurate measure of neural oscillations. To account for this, we used the specparam library (see Methods for details) to examine and remove the exponent and offset from the periodic signal across the three exploration conditions. The results are plotted in Figure 3. For the exponent, a repeated measures ANOVA with factors electrode (Fz, Cz, and Pz) and condition (familiarization, novel and familiar) revealed a main effect of condition (F(2,104) = 6.99, *p* = 0.001, ŋ2 = 0.03) but no interaction (F(2,104) = 1.4, *p* = 0.24, ŋ2 < 0.01). To explore the significant main effect of condition, we conducted three pairwise contrasts: novel versus familiar, novel versus familiarization, and familiar versus familiarization across all three electrode sites. The repeated measures ANOVA with condition (novel, familiar) and electrode (Fz, Cz, and Pz) revealed no main effect of condition (F(1,52) = 0.20, *p* = 0.65, ŋ2 < 0.01). In contrast, the repeated measures ANOVA with condition (novel, familiarization) and electrode did reveal a significant main effect of condition (F(1,52) = 12.44, *p* < 0.001, ŋ2 = 0.04) with a larger exponent for the novel than familiarization condition, and also the contrast familiar versus familiarization revealed a main effect of condition (F(1,52) = 9.00, *p* = 0.004, ŋ2 = 0.03), with a larger exponent for the familiar than familiarization condition. The repeated measures ANOVA for the offset did not reveal any significant results. Although there was an uncorrected main effect of condition, this *p* value did not exceed the critical *p* value as obtained through the permutation testing. Given that we observed significant differences in aperiodic components across the three conditions, we decided to further analyze the data with the aperiodic component removed (i.e., the flattened power spectrum). Beyond this removal to ensure that any differences we find in specific frequency bands are not confounded by aperiodic differences, the aperiodic differences will not be further considered, especially considering that prior research has shown that the aperiodic trend in the EEG is confounded with a similar trend that is found in cardiac data (Schmidt et al. 2024), making a substantive interpretation challenging.

The results of the EEG navigation data are displayed in Figure 2B. We first examined differences in theta power across the three conditions: familiarization, novel, and familiar. A repeated measures ANOVA with factors electrode (Fz, Cz, and Pz) and condition (familiarization, novel, and familiar) revealed a significant interaction effect (F(4,208) = 3.5, *p* = 0.024, ŋ2 = 0.006), although this *F*‐value did not exceed the critical *F*‐value as obtained through the permutation testing. However, since we a priori predicted higher theta power in the familiar compared to the novel condition (based on Kaplan et al. 2012), we ran an exploratory analysis to follow‐up on this interaction effect by comparing the three conditions at each electrode site. This revealed a significant main effect of condition for the frontal electrode site (Fz; F(2,104) = 5.6, *p* < 0.01, ŋ2 = 0.026) and central electrode site (Cz; F(2,104) = 3.3, *p* = 0.04, ŋ2 = 0.016). No significant main effect of condition was found on the posterior electrode site (Pz) (F(2,104) = 0.52, *p* = 0.59, ŋ2 < 0.01). Post hoc *t*‐tests indeed confirmed that exploring a familiar versus novel environment elicited higher theta power over frontal (*t*(52) = −2.42, *p* = 0.018, Cohen's *D* = 0.29) and central (t(52) = −2.32, *p* = 0.024, Cohen's D = 0.31) electrode sites. Furthermore, exploration of a familiar environment compared to exploring an environment for the first time (familiarization) showed higher theta power in frontal electrodes only (*t*(52) = −2.93, *p* = 0.005, Cohen's *D* = 0.37).

### 3.3 Relationship Roaming Entropy & Theta Power During Exploration

We examined the relationship between roaming entropy and theta power for all three exploration conditions by conducting across subject Spearman correlations between the roaming entropy for each condition (familiarization, novel and familiar) and the theta power during that exploration condition (i.e., familiarization, novel and familiar). For the novel condition, we found a significant correlation between roaming entropy and theta power in frontal (*r* = 0.40, *p* = 0.013) and central (*r* = 0.37, *p* = 0.024) electrode sites. No significant correlations were obtained for the familiarization and familiar conditions. Because correlations were computed across multiple electrode sites, we additionally applied a max‐T permutation correction procedure to control for multiple comparisons while accounting for dependency between electrodes. After this correction, the correlations in the novel condition did not remain statistically significant. However, we detected three outlier values in the roaming entropy during exploration of a novel environment (more than 4 SDs from the mean). When removing these outliers, we still found a significant correlation in the novel condition across both frontal (*r* = 0.38, *p* = 0.027; see Figure 2B) and central (*r* = 0.43, *p* = 0.01) electrode sites and no significant correlations were found for the familiarization and familiar conditions (all ps > 0.05). Nevertheless, these correlations likewise did not survive the max‐T permutation correction and should therefore be interpreted with caution.

### 3.4 EEG Episodic Memory Task Results

**FIGURE 4:**
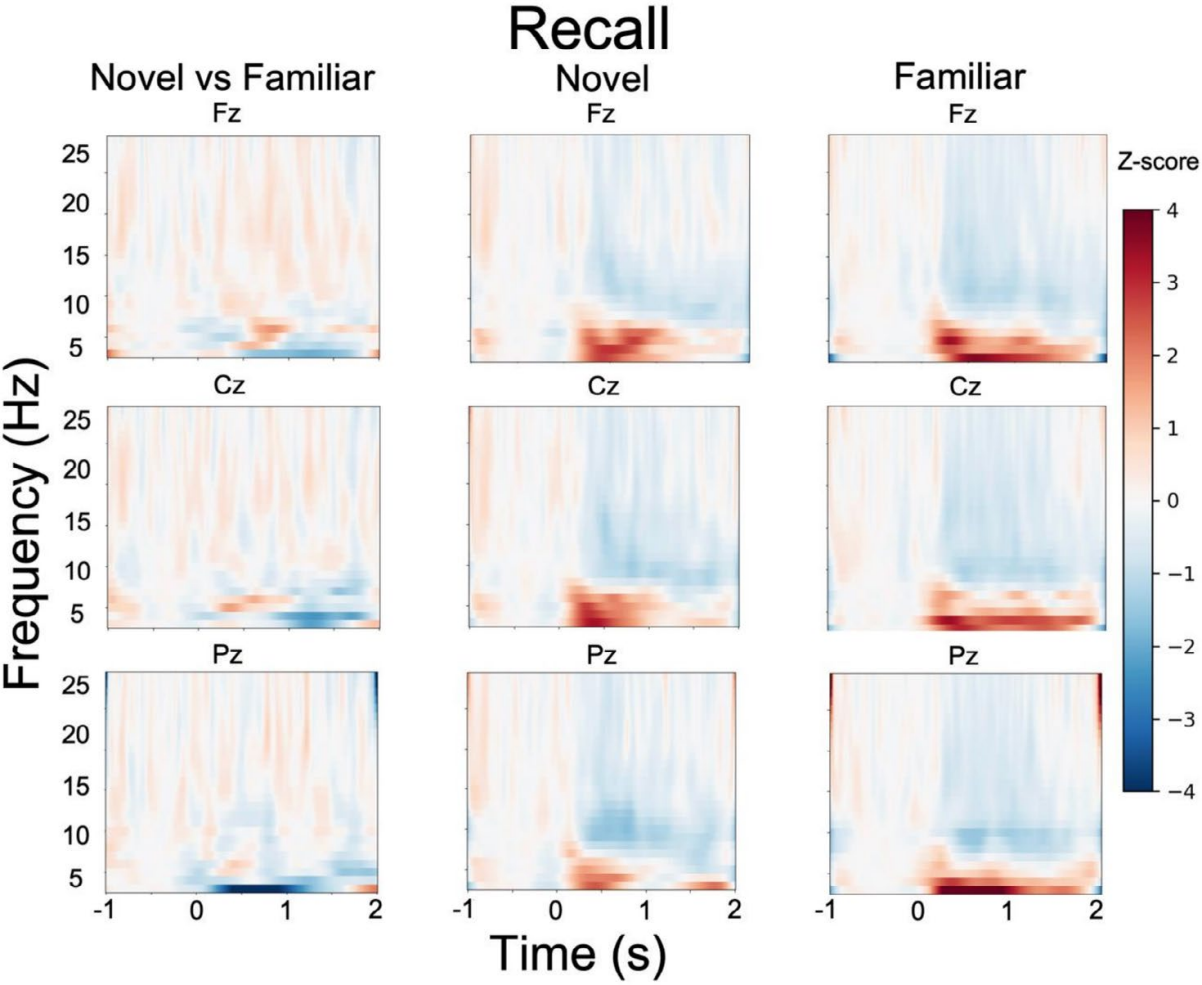
Novel versus familiar successful encoding of subsequent free recall.

**FIGURE 5:**
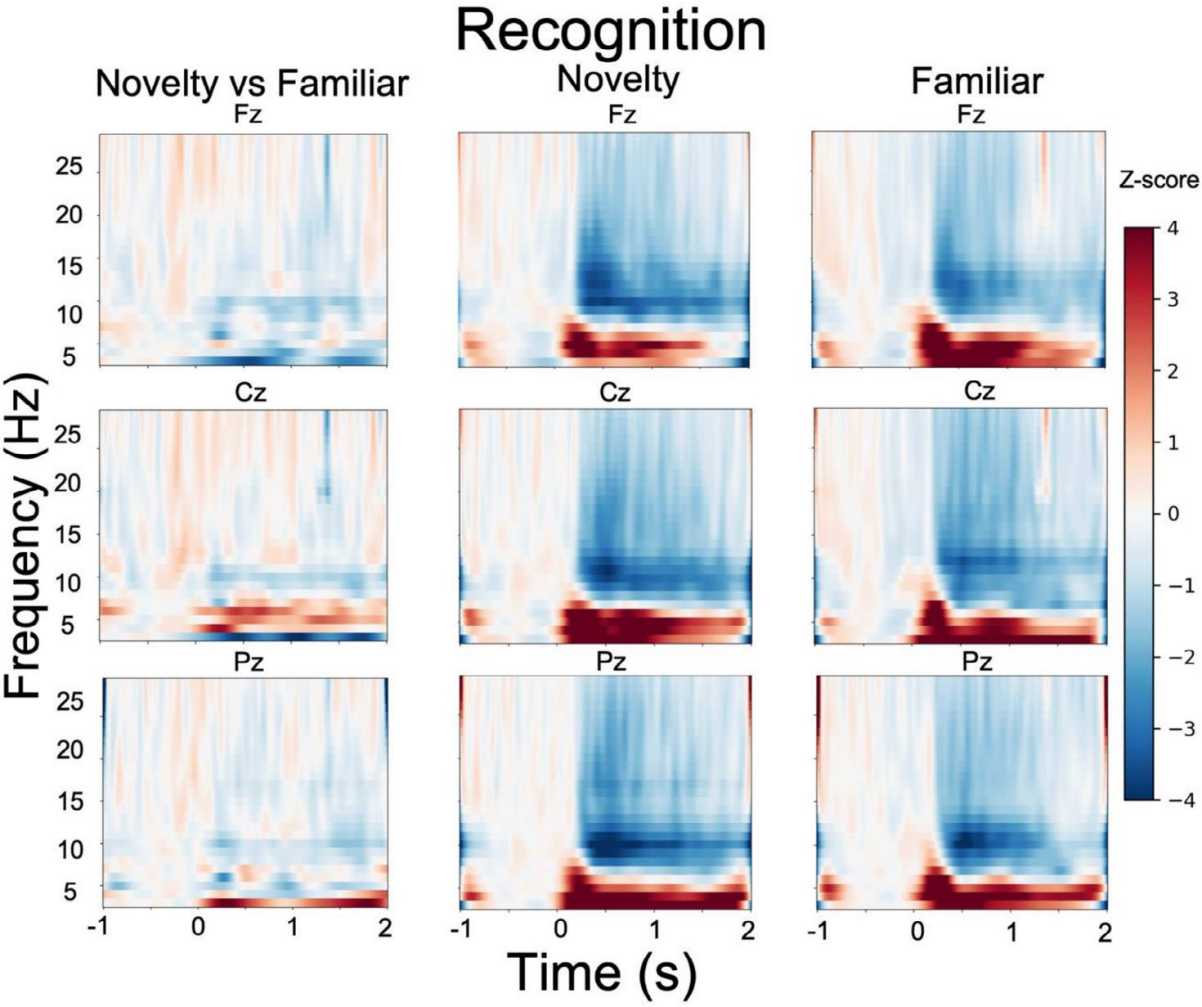
Novel versus familiar successful encoding of subsequent recognition.

Time‐frequency analysis of the task data focused on successfully encoded trials for the novel and familiar conditions. The results are displayed in Figures 4 and 5. We analyzed EEG data based on successful encoding of subsequently recalled (i.e., from the free recall test; displayed in Figure 4) as well as subsequently recognized (i.e., from the old/new recognition memory test; displayed in Figure 5) items. During free recall, several participants had too few trials (i.e., < 10), making a within‐subject analysis infeasible. We therefore conducted a between‐subject analysis of the EEG data, comparing participant groups (e.g., novel vs. familiar condition) rather than regressing small subsamples against behavioral outcomes. For free recall, a 2 by 3 repeated measures ANOVA for theta power in the 200–1000 ms time window with the between‐subjects factors condition (novel, familiar) and within‐subject factor electrode site (Fz, Cz, Pz) revealed a significant main effect of electrode site (*F*(2,150) = 4.13, *p* = 0.017, *ŋ* ^*2*^ = 0.05), but no main effect of condition (*F*(1,150) = 1.13, *p* = 0.29, *ŋ* ^*2*^ < 0.01) nor an interaction (*F*(2,150) = 0.44, *p* = 0.64, *ŋ* ^*2*^ < 0.01). For recognition memory, a 2 by 3 repeated measures ANOVA for theta power in the 200–1000 ms time window with the within‐subject factors condition (novel, familiar) and electrode site (Fz, Cz, and Pz) revealed no main effect of condition (F(1,43) = 1.24, *p* = 0.27, ŋ2 < 0.01), electrode site (F(2,86) = 2.32, *p* = 0.11, ŋ2 < 0.01) or interaction (F(2,86) = 2.1, *p* = 0.13, ŋ2 < 0.01). These EEG results indicate there was no significant difference in theta power between the novel and familiar conditions during the successful encoding of words.

## 4 Discussion

In the current EEG study, we aimed to investigate the benefits of spatial novelty on episodic memory for unrelated information studied briefly after exploration of a virtual environment. Specifically, we sought to investigate and integrate two complementary perspectives: the behavioral mechanisms of exploration and the neural oscillatory processes supporting memory formation. On a behavioral level, we examined whether individual differences in exploration behavior modulate the beneficial effects of novelty on subsequent memory. On a neural level, we investigated whether theta oscillations, often linked as a marker of hippocampal learning dynamics, reflect these behavioral differences during both spatial navigation and episodic encoding. Participants were first familiarized with one VE (day 1). They subsequently explored the same (familiar condition) or a novel environment, one on the same day (day 1) and the other on the subsequent day (day 2; order of novel/familiar conditions counterbalanced across participants). After exploring the novel or familiar environment, participants performed a word learning task. Word recall and word recognition were tested in an immediate and a delayed memory test (±24 h later; day 2 and day 3). Considering the role of theta oscillations, we found a significant increase in theta power in frontal electrodes when participants explored a familiar versus novel environment. Our results also revealed that explorative behavior in the virtual environment, as measured by roaming entropy, was positively correlated with theta power in a frontal and a central electrode for the novelty condition only. Regarding the effect of novelty on memory, overall, no significant differences in free recall for a list of words studied after exploring a novel versus familiar environment were observed. These null findings were corroborated by the EEG results that showed no differences in the theta band during successful encoding of words after exploring a novel versus familiar environment. However, a moderation analysis revealed a significant main effect of novelty as well as a significant interaction effect of novelty and roaming entropy on immediate free recall, indicating that accounting for individual differences in exploratory behavior (measured by roaming entropy) may be an important factor to consider when examining the novelty induced benefits on episodic encoding. We discuss the implications of these results for understanding how novelty and exploration jointly contribute to episodic memory formation and outline directions for future research.

The first part of our analysis focused on theta oscillations during the initial familiarization, and during the exploration of the familiar or novel environment. Consistent with prior work, we found in all three exploration conditions that frontal theta power was increased while navigating a virtual environment (Chrastil et al. 2022; Du et al. 2023). Importantly, exploring an environment that is familiar elicited a theta power increase compared to the exploration of a novel environment or familiarization (i.e., the first round of exploration), whereas the correlation between theta power and the amount of exploration (indexed by roaming entropy) was only significant in the novelty condition. This pattern implies that theta oscillations during spatial exploration may contain multiple sources of information. Theta power has been associated with multiple cognitive functions that may be relevant in this case, including memory processes (see, e.g., Seger et al. 2023), exposure to novelty (see, e.g., Demiralp et al. 2001; Lee et al. 2014), and navigational behavior (see, e.g., Epstein et al. 2017; Kropff et al. 2021). On the one hand, the increased theta power in the familiar compared to the familiarization and novel condition may reflect a memory induced signal since in the familiar condition participants potentially navigated a similar path and saw similar objects as during the familiarization condition. This result is in line with previous work that highlighted (hippocampal) theta power as an important source of navigational memory (Seger et al. 2023). Furthermore, this finding is consistent with results obtained by Kaplan et al. (2012), who observed increased theta power during movement initiation in a familiar versus a novel virtual environment.

The main focus of this paper was to examine the novelty induced benefits on episodic encoding and to what extent exploratory behavior (as measured by roaming entropy) could moderate this novelty effect. We found that free recall of words studied after exploring a novel environment was not significantly better than for words studied after exploring a familiar environment. This null‐result was supported by a Bayesian analysis to confirm evidence for the null‐hypothesis (See Supporting Information). A lack of a main effect of novelty is contradicting with earlier findings of novelty induced memory effects (Abrahan et al. 2020; Ballarini et al. 2013; Baumann et al. 2020; Fenker et al. 2008; Ramirez Butavand et al. 2020; Ruitenberg et al. 2022; Schomaker et al. 2014; Schomaker and Wittmann 2021), yet aligns with other recent studies that also failed to obtain evidence for this novelty effect (Biel et al. 2020; Quent and Henson 2022; Raza et al. 2023; Servais et al. 2026). Furthermore, contrary to what we predicted, the EEG analysis of subsequent memory effects for encoding words in the novel compared to the familiar condition did not reveal any differences in the theta frequency band. These EEG results thus align with the behavioral data where no main effect of novelty on free recall was observed. Interestingly, however, roaming entropy moderated the relationship between condition (novel, familiar) and free recall on the same day. The moderation analysis showed that controlling for variance in roaming entropy does reveal a main effect of novelty on free recall, which, however, must be considered in the context of the significant interaction. Whereas for low roaming entropy values there was a memory benefit of the novel environment, high values of roaming entropy were associated with improved recall in the familiar environment. As visible in Figure 2A, this effect appears to be due to a link between roaming entropy and free recall in the familiar condition, whereas such an association is absent for the novel condition. A potential explanation for this pattern could lie in an unintended novelty effect in the familiar condition, for example when participants explored parts within a familiar environment that they did not explore during the familiarization round. In a novel environment, independent of the extent of exploration, every path that is traveled is novel, meaning that a high or low extent of exploration (indexed by roaming entropy) does not result in substantially different degrees of novelty exposure and may therefore not differentially impact memory. In contrast, in the familiar condition, greater exploration compared to the initial visit likely involves discovering “novel” areas within the familiar environment. As a result, increased exploration in the familiar condition may effectively act as a novelty effect, leading to a positive correlation between exploration and free recall. Importantly, this interpretation also clarifies why a comparable association between roaming entropy and free recall was not observed in the novel condition. In a truly novel environment, all paths are new by definition, such that participants are exposed to novelty regardless of whether their exploration is relatively restricted or extensive. As a result, roaming entropy does not provide additional leverage to differentiate the amount of novelty experienced across individuals in the novel condition, which explains the absence of a correlation between roaming entropy and recall in the novel condition. In contrast, in the familiar condition, variability in roaming entropy directly translates into variability in exposure to previously unexplored areas, making it a meaningful predictor of recall performance. To address this tentative hypothesis that familiar environments could have influenced memory due to remaining unexplored patches, we ran an exploratory analysis for which we calculated the overlap between the path traveled during the initial visit (i.e., familiarization round) and the path traveled during the second exploration round of the same environment. Consistent with the hypothesis that the interaction between the condition (novelty, familiar) and roaming entropy reflects a memory benefit in free recall after increased exposure to novel paths in the familiar condition when exploration behavior (i.e., roaming entropy) is enhanced, the interaction between novelty and roaming entropy was no longer significant when accounting for overlap. This result implies that overlap accounts for some of the variance that was previously attributed to the interaction. A potential interpretation is that for low values of overlap, indicating more exposure to novel rather than familiar paths, there is a relationship between roaming entropy and free recall in the familiar condition, whereas such a relationship is absent when overlap is high. As an additional exploratory test of this mechanism, we also examined whether the change in roaming entropy between the familiarization and familiar exploration phases predicted free recall performance. This analysis revealed a non‐significant association, thereby not supporting our hypothesis that increased exploration in the familiar condition is a form of a novelty effect. It is important to note, however, that we believe that the overlap measure provides a more direct operationalization of “newly explored space” than a difference score in roaming entropy, as roaming entropy itself does not explicitly distinguish between revisiting known areas versus exploring genuinely new ones. As this explanation and analyses were postulated and executed post hoc, future experiments should further test whether increased exploration in the familiar condition effectively acts as a novelty effect.

Our results reveal that exploration behavior in a virtual environment may be an important factor in studying the novelty effect, a new insight that has not be investigated earlier. Some prior research investigating the effect of navigation behavior specifically on spatial memory, however, is somewhat related to the findings we report here. For example, Puthusseryppady et al. (2024) found that in midlife adults (aged 43–61) less spatial exploration is related to poorer spatial memory. The researchers also found that differences in exploration variables like distance traveled and hallway visits partially explained the memory performance gap between midlife and younger adults. For midlife adults, both the quantity and quality of exploration were linked to spatial memory, while for young adults, only exploration quality showed a similar trend (Puthusseryppady et al. 2024). Similarly, a study by Meade et al. (2019) highlighted that active navigation, as opposed to passive observation, led to higher accuracy in spatial memory tasks, and this effect was particularly strong in older compared to younger adults. Together, these studies are in line with the hypothesis that the way in which individuals explore an environment influences subsequent memory of that environment. Considering that the postulated mechanism behind the beneficial memory effects of exploring a novel environment involves the enhancement of a weak memory trace due to another event (i.e., novelty) eliciting a *stronger* memory process (see, e.g., Moncada and Viola 2007), together with the findings that the extent of active navigation is linked to better memory of the navigated environment (Meade et al. 2019; Puthusseryppady et al. 2024), the link between exploration behavior in the familiar condition and immediate recall in our study may be explained by more extensive exploration leading to a stronger engagement of memory systems. This interpretation is consistent with the previously discussed theta power increase in the familiar condition, which may reflect memory processes (see, e.g., Seger et al. 2023).

Our study is the first to show that considering exploration patterns may be particularly relevant when a novel compared to a familiar environment is explored and highlights the importance for future studies to take exploration patterns into account whilst studying the novelty induced memory benefits in episodic memory. However, our current design also has some limitations and there are factors that may be worth considering when conducting future research on the novelty effect. First, in our design, the familiarization and familiar session were sometimes conducted on the same day. We recommend that these sessions be spaced across separate days to ensure that the familiar condition truly reflects recognition memory, as this initial familiarization may itself induce already some kind of “novelty” effect, which should first be properly consolidated before participants explore the environment again to ensure it is genuinely familiar rather than a continuation of novelty‐driven exploration. This is a potential explanation for why we did not observe a general novelty induced benefit on successful encoding of words. Second, our spatial exploration was conducted in 3D virtual space presented on a two‐dimensional screen, however exploration in an immersive virtual reality (VR) environment may be more effective and would better align with animal studies where subjects actively navigate or explore their surroundings, potentially enhancing the impact of novelty. Third, the timing of memory testing relative to novelty exposure requires further investigation. While animal studies suggest that novelty effects emerge 24 h later, human studies have reported effects as early as 15 min post‐exploration. In line with Quent and Henson (2022), our study found no novelty‐induced memory benefits on delayed recall intervals, suggesting that timing may play a critical role. Fourth, the stimulus type and task selection should be carefully considered. Word‐based tasks may be more susceptible to interference, whereas image‐based tasks or associative memory tasks, more reliant on hippocampal function, might better capture novelty effects, considering that it seems to be specifically the performance on hippocampus‐dependent learning tasks that is improved when accompanied by novelty (Lorents et al. 2023). Relatedly, the beneficial effects of novelty on memory might be particularly present for weakly encoded material, as was previously shown in the animal literature as well as for emotion‐related retroactive enhancement of memory in humans (Ballarini et al. 2013; Dunsmoor et al. 2022). Although the performance on the recall and recognition tests in this study does suggest that the encoding of the material was not particularly strong, the novelty effect may be more apparent when using a shallower encoding task (e.g., judging whether the word starts with a vowel or consonant) rather than the one used here (living versus non‐living). Fifth, the magnitude of novelty effects may vary across age groups, with potentially stronger effects in children and adolescents than in adults and older individuals, as observed in Schomaker and Wittmann (2021) as well as Ballarini et al. (2013). Since only adults participated in this study, weaker effects in this age group may have influenced our findings. Sixth, future studies could also examine alpha oscillations during the recognition phase, as our time–frequency representations suggested possible alpha modulations following exploration of novel environments. Investigating these effects could provide additional insights into how novelty influences memory retrieval processes. Finally, future studies should explore the distinction between proactive (i.e., before encoding) and retroactive (after encoding) novelty exploration, as recent findings indicate that retroactive novelty exposure may not reliably induce memory benefits (Quent and Henson 2022; Lorents et al. 2023, Raza et al. 2023, preprint).

Taken together, we did not observe a general novelty‐induced memory benefit on free recall, a finding that was corroborated by the lack of theta power differences associated with successful encoding of words after exploring a novel versus familiar environment. Accounting for variance in spatial exploration patterns revealed a main effect of novelty as well as an interaction between roaming entropy and recall such that a beneficial effect of increased exploration on free recall was observed in the familiar but not in the novel condition. Our findings underscore the importance of incorporating spatial behavior when analyzing novelty‐related effects on episodic memory. By addressing the considerations outlined above (spacing sessions, using immersive VR, and finetuning the timing between novelty exposure and the learning events), future research can refine our understanding of how novelty influences episodic memory and identify the optimal conditions for enhancing memory through novelty exposure.

## Author Contributions

Conceptualization: David A. Vogelsang and Judith Schomaker; Methodology: David A. Vogelsang and Judith Schomaker; Formal analysis: David A. Vogelsang, Carla Maló Calmus, and Judith Schomaker; Investigation: David A. Vogelsang, Carla Maló Calmus, Madeleine Vohs, Kreeta I.L. Kerkkänen, Svetlin Hansov, and Judith Schomaker; Writing – original draft preparation: David A. Vogelsang and Judith Schomaker; Writing – review and editing: David A. Vogelsang, Carla Maló Calmus, Madeleine Vohs, Kreeta I.L. Kerkkänen, Svetlin Hansov, and Judith Schomaker; Resources: David A. Vogelsang and Judith Schomaker; Supervision: David A. Vogelsang and Judith Schomaker.

## Conflicts of Interest

The authors declare no conflicts of interest.

## Supporting information


**Data S1:** Supporting Information.

## Data Availability

The data that support the findings of this study are available from the corresponding author upon reasonable request.
