# Supplementary material for: Neural and Navigational Features Influencing the Novelty Induced Benefits on Episodic Memory
Source: Hippocampus. 2026 Jun 18;36(4):e70109. doi: 10.1002/hipo.70109 (PMC13277633; doi:10.1002/hipo.70109)
Supplement: Supplementary file 1 — Data S1: Supporting Information. [file HIPO-36-0-s001.docx]

**Supplementary Materials**

*S1. Ratings*

*Ratings and questionnaire analyses*

The effect of session on motivation, arousal and presence was investigated with separate repeated-measures ANOVAs with session (1; 2; 3) as a within-subjects factor. As motivation was expected to decrease over the course of the study, a main effect was followed up with a linear contrast. The difference between the novel and familiar conditions was investigated with a paired-samples t-test.

*Ratings and questionnaires: Motivation, arousal, and presence*

The effect of session on motivation was investigated with a repeated-measures ANOVA with session (1; 2; 3) as a within-subjects factor. Session affected motivation, F(2,104) = 3.45, p = .036, ŋ2 = .062. A follow-up linear contrast suggested that motivation linearly decreased over sessions, F(1,52) = 1312.37, p < .001, ŋ2 = .962. No difference was found between motivation in the novel versus the familiar condition, t(52) = .394, p = .695.

Similarly, the effects of session and novelty were investigated for arousal. A main effect of session was observed, F(2,104) = 3.33, p = .040, ŋ2 = .060, however, no linear decrease of session was found for arousal, F(1,52) = 4.44, p = .040, ŋ2 = .079. Nor were differences in arousal observed between the novel and familiar conditions, t(52) = 1.01, p = .314.

For presence no main effect of session was observed, F(1,52) = 0.97, p = .383, ŋ2 = .018, nor was difference found between the novel and familiar conditions, t(52) = 0.27, p = .787.

*S2. Order Effects*

Effects including the factor order, were further investigated with follow-up repeated-measures ANOVAs per order (Green-Pink-Green; Green-Pink-Pink; Pink-Green-Pink; Pink-Pink-Green) with time (immediate; delayed) and novelty (novel; familiar) as within-subjects factors.

*Word Analysis Recall*

For the order Pink-Pink-Green there was a main effect of time, with higher recall for immediate than delayed memory, F(1,17) = 11.59, p = .003, ŋ2 = .405 and no main effect of novelty, F(1,17) = 2.46, p = .136, ŋ2 = .126. However, an interaction between novelty and time was observed, F(1,17) = 10.17, p = .005, ŋ2 = .374, with better memory for the novel than familiar condition for immediate memory, and better memory for the familiar rather than novel condition for delayed memory.

For the order Green-Pink-Pink there was a main effect of time, with higher recall for immediate than delayed memory tests, F(1,19) = 19.85, p < .001, ŋ2 = .511. Also a main effect of novelty was observed, with higher recall for the novel than familiar condition, F(1,19) = 5.74, p = .027, ŋ2 = .232. For this order, no interaction between novelty and time was observed, F(1,19) = 0.293, p = .594, ŋ2 = .015.

For the order Pink-Green-Pink a main effect of time was observed, with higher recall for immediate than delayed memory, F(1,13) = 12.26, p = .004, ŋ2 = .485. No main effect of novelty was found, F(1,13) = 0.40, p = .844, ŋ2 = .003. However, an interaction between novelty and time was observed, F(1,13) = 5.98, p = .030, ŋ2 = .315, with better memory for the familiar than novel condition for immediate, and better memory for the novel rather than familiar condition for delayed memory (note, this is the opposite of the effect observed in the Pink-Pink-Green condition.

In the Green-Pink-Green order condition no main effect of time was found, F(1,3) = 5.36, p = .104, ŋ2 = .641, nor a main effect of novelty, F(1,3) = 3.39, p = .163, ŋ2 = .531. An interaction between novelty and time was observed, F(1,3) = 24.02, p = .016, ŋ2 = .889, with higher recall for the familiar rather than novel condition for immediate memory, and higher recall for the novel than familiar condition for delayed memory. Note, however, that this condition only included four participants, and interpretation of these results should be cautious.

*Bayesian statistics*

To further investigate the null effect of novelty and the null effect of the interactions between novelty and other factors (i.e., time and order) in our main ANOVA, we employed a Bayesian statistics approach. The results of these analyses are reported in S3. We compared five models that included the factor novelty and the two-way, and three-way interactions of novelty with other factors (i.e., model 1. only novelty, 2. novelty + novelty*time, 3. novelty + novelty*time + novelty*order, 4. novelty + novelty*order + order*time, and 5. novelty + novelty*time + novelty*order + novelty*time*order) to a null model that included only the factors of time and order, and their interaction.

For model 1 (novelty) versus the null model (novelty versus null model including time, order and time*order) we observed a Bayes Factor (BF01) of 4.12, indicating that our observed findings are approximately 4.12 times more likely under the null model, providing moderate support for the null model.

For model 2 (novelty + novelty*time) versus the null model we found that the BF01 was 20.40, suggesting that the null model was approximately 20.40 times more likely than model 2, providing strong support for the null model.

For model 3 (novelty + novelty*time + novelty*order) versus the null model we observed a BF01 of 3.12, suggesting that the findings are 3.12 times more likely under the null hypothesis, providing moderate support for the null model.

For model 4 (novelty + novelty*order + order*time) we observed a BF01 of 0.74, suggesting that the findings are approximately 1.35 times (BF10 = 1/0.74 = 1.35) more likely under the alternative model 4, than under the null model. However, since this BF01 is relatively close to 1 there no strong evidence in favor of either model.

Finally, for model 5 (novelty + novelty*time + novelty*order + novelty*time*order) we observed a BF01 of .13, suggesting that our word recall findings are approximately 7.75 times (BF10 = 1/0.13 = 7.75) more likely under the alternative model 5, than under the null model, indicating moderate evidence in favor of the alternative model. These findings suggest that when we include the experimental variables novelty and time, and the between-subjects variable order, and their interactions, this model could explain the findings better than the null model.

*Exploration and landmark memory*

Exploration behaviour was investigated with roaming entropy. A statistical trend effect for higher roaming entropy in the novel (mean RE = .017, SD = .057) compared to the familiar VE (mean RE = .002, SD = .001) was observed, t(38) = 1.91, p = .064.

As expected, participants had a higher corrected hit rate for landmarks from the familiar environment that they explored twice, rather than the novel environment that they only explored once, t(51) = 6.48, p < .001.

*EEG Analysis*

We also ran an analysis to examine the effect of order (which refers to the order of how the environments were presented) on theta power for novelty versus familiar subsequently recalled words. The orders we presented were: pink-pink-green, pink-green-pink, green-pink-pink, green-pink-green. The aim of this analysis was to examine whether the order of environments could influence the novelty effect. For each order effect, we examined differences in theta power in a 2 by 3 repeated measured ANOVA with factors condition (novel, familiar) and electrode (frontal, central, posterior). The results indicated that none of the order showed a significant main effect of condition nor a significant condition by electrode interaction: green-pink-pink: condition main effect F = .08, p = .78; interaction F = .13, p = .87; pink-green-pink condition main effect F=,1.71 p=0.22, interaction F = .1, p = .91; pink-pink-green condition main effect: F = .006, p = .94, interaction F = 2.11, p = .14.

*S3. Bayesian statistics*

To further investigate the null effect of novelty and the null effect of the interactions with novelty in our main ANOVA, we employed a Bayesian statistics approach. We compared five models that included the factor novelty (1. only novelty) and the two-way and three-way interactions of novelty with other factors (2. novelty + novelty*time, 3. novelty + novelty*time + novelty*order, 4. novelty + novelty*order + order*time, and 5. novelty + novelty*time + novelty*order + novelty*time*order) to a null model that included only the factors of time and order, and their interaction.

For model 1 (novelty) versus the null model (novelty versus null model including time, order and time*order) we observed a Bayes Factor (BF01) of 4.12, indicating that our observed findings are approximately 4.12 times more likely under the null model, providing moderate support for the null model.

For model 2 (novelty + novelty*time) versus the null model we found that the BF01 was 20.40, suggesting that the null model was approximately 20.40 times more likely than model 2, providing strong support for the null model.

For model 3 (novelty + novelty*time + novelty*order) versus the null model we observed a BF01 of 3.12, suggesting that the findings are 3.12 times more likely under the null hypothesis, providing moderate support for the null model.

For model 4 (novelty + novelty*order + order*time) we observed a BF01 of 0.74, suggesting that the findings are approximately 1.35 times (BF10 = 1/0.74 = 1.35) more likely under the alternative model 4, than under the null model. However, since this BF01 is relatively close to 1 there no strong evidence in favor of either model.

Finally, for model 5 (novelty + novelty*time + novelty*order + novelty*time*order) we observed a BF01 of .13, suggesting that our word recall findings are approximately 7.75 times (BF10 = 1/0.13 = 7.75) more likely under the alternative model 5, than under the null model, indicating moderate evidence in favor of the alternative model. These findings suggest that when we include the experimental variables novelty and time, and the between-subjects variable order, and their interactions, this model could explain the findings better than the null model.
